# Routine Assessment of Patient Index Data 3 (RAPID3) alone is insufficient to monitor disease activity in rheumatoid arthritis in clinical practice

**DOI:** 10.1136/rmdopen-2019-001050

**Published:** 2019-11-28

**Authors:** Niels W Boone, Alexandre Sepriano, Paul-Hugo van der Kuy, Rob Janknegt, Ralph Peeters, Robert B M Landewé

**Affiliations:** 1 Department of Clinical Pharmacy, Pharmacology and Toxicology, Zuyderland Medical Centre Sittard-Geleen, Sittard-Geleen, Limburg, The Netherlands; 2 Department of Rheumatology, Leiden University Medical Center, Leiden, Zuid-Holland, The Netherlands; 3 NOVA Medical School, Universidade Nova de Lisboa, Lisbon, Portugal; 4 Department of Clinical Pharmacy, Erasmus MC, Rotterdam, Zuid-Holland, The Netherlands; 5 Department of Rheumatology, Zuyderland Medical Centre Heerlen, Heerlen, Limburg, The Netherlands; 6 Department of Rheumatology, Amsterdam University Medical Centres, Amsterdam, Noord-Holland, The Netherlands

**Keywords:** Rheumatoid arthritis, disease activity, DAS28, RAPID3, responsiveness, patient reported outcome measure (PROM)

## Abstract

**Objective:**

To test the longitudinal association between patient-reported outcome, Routine Assessment of Patient Index Data 3 (RAPID3) and the Disease Activity Score in 28 joints that includes the erythrocyte sedimentation rate (DAS28-ESR) in routine-care patients with rheumatoid arthritis (RA).

**Methods:**

Patients with RA treated with disease-modifying antirheumatic drugs were included in this prospective observational cohort. The longitudinal association between RAPID3 (0–10) and DAS28-ESR and its individual components (swollen joint count (SJC), erythrocyte sedimentation rate (ESR) (mm/hour), tender joint count (TJC) and patient global assessment (PGA)) was tested using generalised estimating equations in patients with more than two consecutive visits with data on RAPID3 and DAS28-ESR. Interactions between RAPID3 and gender, pain, PGA and age at baseline were tested, and if significant (p<0.20) and clinically relevant, models were fit in the corresponding strata.

**Results:**

In total, 330 patients were included (mean follow-up 10.7 (SD 9.7) months, female gender 67.9%). The longitudinal association between RAPID3 and DAS28-ESR was weak (β=0.29 (95% CI 0.24 to 0.35), n=207), meaning that one unit increase in RAPID3 corresponded to a 0.29 unit increase in Disease Activity Score in 28 joints (DAS28). RAPID3 was most strongly associated with subjective (TJC: β=0.89 (95% CI 0.61 to 1.17); PGA: β=0.94 (95% CI 0.84 to 1.04)) and not with objective components of DAS28 (SJC: β=0.29 (95% CI 0.17 to 0.41), n=172). The association between RAPID3 and ESR was poor but modified by gender, being only significant in men (β=0.37 (95% CI 0.08 to 0.67)).

**Conclusions:**

These data suggest that RAPID3 does not sufficiently capture changes in objective inflammatory signs. Monitoring by RAPID3 alone is therefore insufficient to follow disease activity in patients wth RA in clinical practice.

**Key messages**

**What is already known about this subject?**

- The Routine Assessment of Patient Index Data 3 (RAPID3) is considered one of the best validated patient-reported outcome measures in rheumatoid arthritis (RA).

**What does this study add?**

- RAPID3 is poorly associated with Disease Activity Score in 28 joints that includes the erythrocyte sedimentation rate and especially with its objective components, swollen joint count and erythrocyte sedimentation rate, over time in patients with RA followed up in daily practice and stable on treatment.
- RAPID3 associates very well with the subjective components of Disease Activity Score in 28 joints, tender joint count and patient global assessment, which confirms that RAPID3 is strongly driven by subjective pain instead of inflammatory disease activity.

**How might this impact on clinical practice?**

- RAPID3 alone is insufficient to capture objective signs of inflammation in routine-care patients with RA.
- The sole use of subjective patient monitoring instruments can be misleading and potentially lead to overtreatment of patients with RA, especially in the absence of convincing signs of inflammation.

## Introduction

Patient-reported outcome measures (PROMs) yield clinically important information and have been used for many years in the development and evaluation of medical interventions.^1–4^ These measures primarily aim at reflecting patients’ unique perspectives and, as such, can contribute to engage patients, clinicians and other stakeholders (eg, government and payers) in judging the relevance of treatment effects and the development of value-based healthcare.^5 6^

PROMs have been awarded a prominent place in the management of rheumatic and musculoskeletal diseases such as rheumatoid arthritis (RA). For instance, the patient global assessment (PGA) has been included as one criterion to define the American College of Rheumatology (ACR)–EULAR Boolean definition of remission.^3 7 8^ Also, both ACR and EULAR prescribe the use of patient-driven constructs as part of the core set to measure disease activity in daily practice as well as clinical research.^3 4^

One example of a composite disease activity PROM is the Routine Assessment of Patient Index Data 3 (RAPID3), which is considered the best validated PROM in RA.^9 10^ In contrast to the Disease Activity Score in 28 joints that includes the erythrocyte sedimentation rate (DAS28-ESR) and the presence of swelling in 28 joints, RAPID3 is solely based on three (subjective) patient-reported domains: physical function, pain and PGA. Thus, it has been suggested that RAPID3 (similar to other PROMs) could mostly translate factors other than inflammation, such as fatigue, depression or even symptoms driven by comorbidities.^11 12^ In reality, the question if whether or not RAPID3 truly captures inflammation-driven disease activity in clinical practice is still unanswered.^10^ If not, this means that if RAPID3 is used to guide treatment decisions on drugs mainly targeting inflammation, clinicians may expose patients to unnecessary risks with only little or no benefit.

We investigate here the longitudinal relationship between RAPID3 and DAS28-ESR (including its individual components) in patients with RA in order to determine RAPID3’s ability to monitor changes in inflammation-driven disease activity appropriately.

## Patients and methods

### Patients and study design

Patients with a clinical diagnosis of RA (and fulfilling the ACR 1987 classification criteria^13^) were included in this prospective observational study performed in a large rheumatology unit in the Netherlands. Patients could have been treated with conventional synthetic disease-modifying antirheumatic drugs (csDMARDs) and/or biological disease-modifying antimodifying antirheumatic drugs (bDMARDs) according to their treating rheumatologists and were followed up every 3 months for a up to 2 years (April 2013–April 2016). All patients provided informed consent before inclusion.

### Data collection and disease scores

Patients’ characteristics, including gender, age and disease duration (years), were collected at baseline by rheumatologists and research nurses. Measures of disease activity (DAS28-ESR and RAPID3), laboratory data (erythrocyte sedimentation rate (ESR)) and medication status (bDMARD and/or csDMARD, yes/no) were assessed every 3 months. RAPID3 is composed of three domains: physical function (0–10), pain (Visual Analogue Scale 0–10) and PGA of disease activity (0–10).^9^ The sum of the three domains is expressed as a continuous total score (range from 0 (best) to 30 (worst)). To facilitate comparison with DAS28-ESR, we converted the total score to a 0–10 scale in accordance with the RAPID3 score template.^9^ The total DAS28-ESR (range: 0–9.3)^14^ was computed with four variables: tender joint count (TJC 0–28), swollen joint count (SJC 0–28), PGA (0–10) and ESR.

### Statistical analysis

First, we used generalised estimating equations (GEEs), with time as the explanatory variable of interest, to assess how RAPID3 and Disease Activity Score in 28 joints (DAS28) changed over time (in separate models). Different transformations of time were tested to assess which yielded the best fit (assessed by the quasi-likelihood under the independence model criterion). A non-linear model was chosen if best fitting the data and if the non-linear factor (eg, quadratic term) was statistically significant (p<0.05).

Second, the longitudinal association between RAPID3 (explanatory variable) and DAS28-ESR (main outcome) was tested in GEE models with autoregression (ie, adjusting for the outcome in t-1). The association between RAPID3 and each individual component of DAS28 (SJC, ESR, TJC and PGA) was also tested in separate GEE models. GEE models with autoregression allow for a truly longitudinal interpretation of the association of interest by ‘isolating’ the within-subject effect while correcting for the inherent correlation by specifying a ‘working correlation matrix’. The exchangeable correlation matrix was used since it proved to better fit the data. Interactions between RAPID3 and gender, pain, PGA and age were tested, and if statistically significant (p<0.20) and clinically relevant, the association of interest was tested in stratified models (median value at baseline for continuous variables). In all models, only patients with data on the outcome and explanatory variable available in at least two consecutive visits were included (details on statistical analysis available in online supplementary text S1). All analyses were performed in STATA V.15.1).

10.1136/rmdopen-2019-001050.supp1 Supplementary data

## Results

In total, 330 patients (1348 visits; mean 4.0 (SD 2.7) number of visits, mean 10.7 (SD 9.7) months of follow-up) were included. Baseline characteristics, disease activity parameters and treatment status are shown in table 1.

**Table 1:** Baseline characteristics of included patients

Parameter	Patients with RA (N=330)
Female gender, n (%)	224 (67.9)
Age (years), mean (SD)	62.0 (11.6)
Disease duration, mean (SD)	11.2 (9.6)
DAS28-ESR,* mean (SD)	3.3 (1.4)
SJC (0–28),^†^ mean (SD)	2.3 (3.5)
TJC (0–28),^†^ mean (SD)	4.1 (5.5)
ESR (mm/hour),^†^ mean (SD)	18.7 (17.6)
RAPID3 total score (0–10),^‡§^ mean (SD)	3.8 (2.1)
RAPID3 function (0–10), mean (SD)	2.5 (1.9)
RAPID3 VAS pain (0–10), mean (SD)	4.3 (2.6)
RAPID3 PGA (0–10), mean (SD)	4.7 (2.4)
Treatment status	
bDMARD only,^¶^ n(%)	155 (47)
MTX only, n (%)	77 (23.3)
bDMARD plus MTX, n (%)	71 (21.5)
No bDMARD**/MTX, n (%)	27 (8.2)

*DAS28-ESR (four variables, ESR-based).

†N=213.

‡RAPID3 (functioning, VAS pain and PGA).

§RAPID3 0–30 was converted to a 0–10 scale; high numbers reflect bad scores.

¶bDMARD includes TNFi (81.9%; etanercept, infliximab, adalimumab, certolizumab–pegol and golimumab) and no TNFi (18.1%; rituximab, abatacept and tocilizumab).

**Patients (n=27) neither on bDMARDs nor on MTX were treated with leflunomide, sulfasalazine or hydroxychloroquine.

bDMARD, biological disease-modifying antirheumatic drug; DAS28-ESR, Disease Activity Score in 28 joints that includes the erythrocyte sedimentation rate; ESR, erythrocyte sedimentation rate; MTX, methotrexate; PGA, patient global assessment; RA, rheumatoid arthritis; RAPID3, Routine Assessment of Patient Index Data 3; SJC, swollen joint count; TJC, tender joint count; TNFi, tumor necrosis factor inhibitor; VAS, Visual Analogue Scale.

In our population, both DAS28 and RAPID3 had shown very limited change over time following a linear distribution. On average, DAS28 decreased 0.02 units per month (β=−0.02 (95% CI −0.02 to −0.01)), while RAPID3 increased 0.01 units (β=0.01 (95% CI 0.00 to 0.01)).

### Longitudinal association between RAPID3 and DAS28

**Figure 1:**
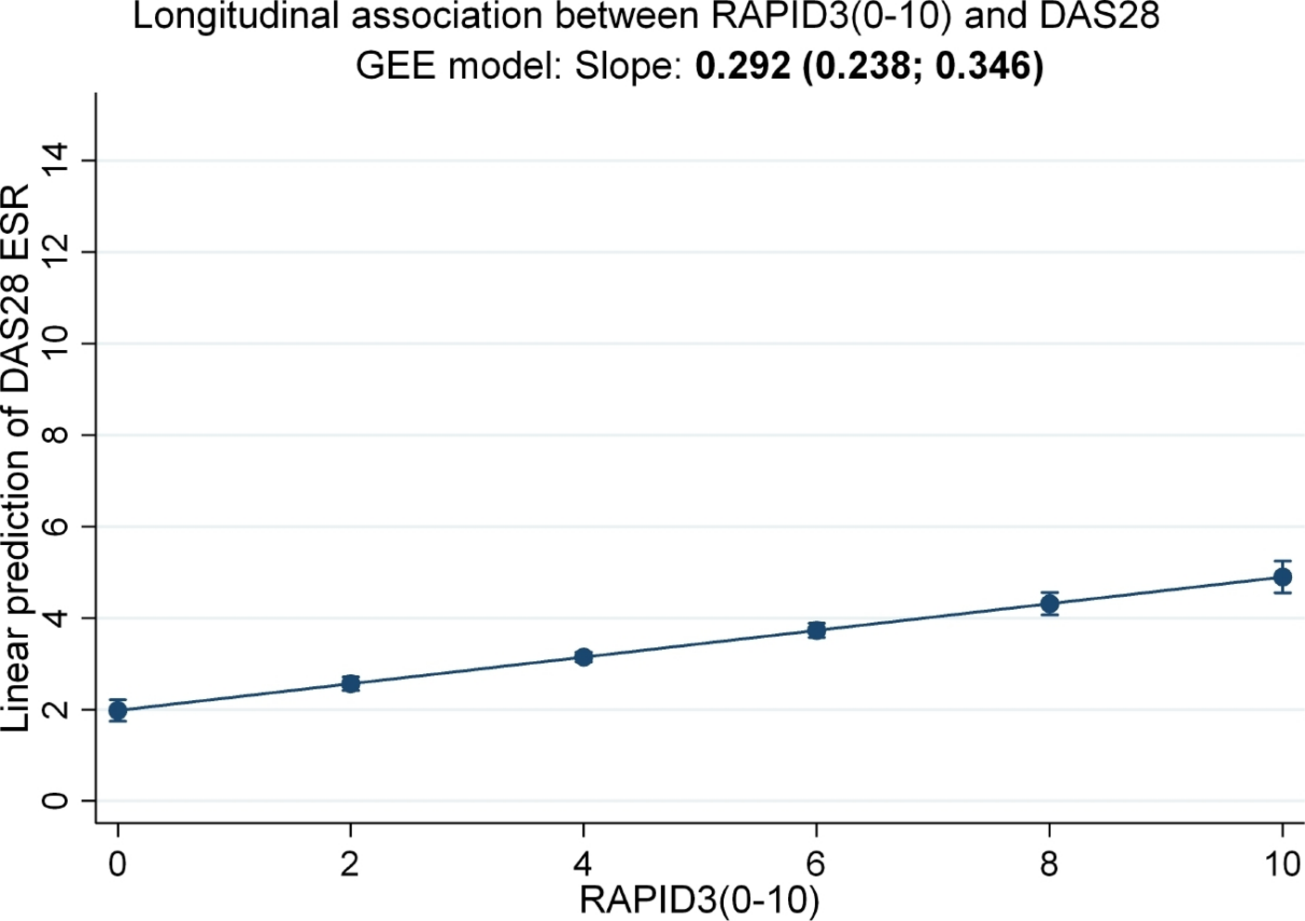
Longitudinal association between RAPID3 and DAS28-ESR over time (N=207). Longitudinal GEE model with autoregression. RAPID3 0–30 converted to a 0–10 scale; high numbers reflect worse outcome. RAPID3 domains: functioning, pain and patient global assessment; DAS28 (four variables, erythrocyte sedimentation rate-based). DAS28, Disease Activity Score in 28 joints; DAS28-ESR, Disease Activity Score in 28 joints that includes the erythrocyte sedimentation rate; GEE, generalised estimating equation; RAPID3, Routine Assessment of Patient Index Data 3.

There was a weak but statistically significant longitudinal association between RAPID3 and DAS28-ESR (β=0.29 (95% CI 0.24 to 0.35), n=207). This means that 1 unit increase in RAPID3 corresponded to only 0.29 unit increase in DAS28 over time (figure 1). The association between RAPID3 and DAS28 individual components (n=172) yielded different results for subjective and objective domains (table 2). The difference in the numbers of patients included between models with DAS28 and models with individual components stems from the fact that the individual components of DAS28 were not available to all patients, although the overall score was.

**Table 2:** Longitudinal association between RAPID3 (0–10) and DAS28-ESR, including individual components

Outcome	DAS28 β (95% CI)^†‡^	SJC β (95% CI)§	TJC β (95% CI)§	PGA β (95% CI)§	ESR β (95% CI)§	
					Male (n=52)	Female (n=122)
RAPID3* (0–10)	**0.29** (0.24 to 0.35)	**0.29** (0.17 to 0.41)	**0.89** (0.61 to 1.17)	**0.94** (0.84 to 1.04)	**0.37** (0.08 to 0.67)	−0.03 (−0.47 to 0.41)

Statistically significant results are shown in bold.

*RAPID3 domains: functioning, pain and PGA.

†DAS28 (four variables, ESR-based).

‡Models derived from 207 patients.

§Models derived from 172 patients.

DAS28, Disease Activity Score in 28 joints; DAS28-ESR, Disease Activity Score in 28 joints that includes the erythrocyte sedimentation rate; ESR, erythrocyte sedimentation rate; PGA, patient global assessment; RAPID3, Routine Assessment of Patient Index Data 3; SJC, swollen joint count; TJC, tender joint count.

RAPID3 was found to be strongly associated with the TJC (β=0.89 (95% CI 0.61 to 1.17)) and with PGA (β=0.94 (95% CI 0.84 to 1.04)). This means that 1 unit increase in RAPID3 corresponds to almost 1 unit increase of PGA. In comparison, the association between RAPID3 and the SJC was weak at best (β=0.29 (95% CI 0.17 to 0.41)). Only the association between RAPID3 and ESR was modified by gender (interaction p value of 0.141). It was somewhat stronger in men (β=0.37 (95% CI 0.08 to 0.67)) than in women (β=−0.03 (95% CI −0.47 to 0.41)). No other significant interactions were found.

## Discussion

In this prospective observational study, we have shown that RAPID3 is poorly associated with DAS28-ESR over time in patients with RA followed in daily practice. This is mainly due to the weak association between RAPID3 and the objective components of DAS28 (SJC and ESR), while RAPID3 associates very well with DAS28 subjective components (TJC and PGA).

The association between RAPID3 and DAS28, including the individual components of DAS28, have been previously investigated but, thus far, only in cross-sectional observational studies.^15–17^ Our findings are mostly in line with these studies, which have shown a weak to moderate association between the two scores. Our study adds to the existing literature by showing that this association is also absent in a longitudinal setting, thus increasing internal validity, and in patients followed up in routine clinical practice (external validity). Despite the lack of association between RAPID3 and DAS28 in observational studies, RAPID3 and other PROMs have been consistently shown to discriminate well regarding responses between active treatment and placebo in randomised clinical trials (RCTs).^18 19^ Although these data strongly argue in favour of PROMs to detect treatment effects in trials, these do not necessarily support their use in daily clinical practice. In fact, we have recently shown, using data from the same cohort, that patients treated with both bDMARDs and methotrexate did not have different RAPID3 scores over time compared with those on bDMARDs alone, while a difference could have been clearly seen with DAS28 (especially with SJC).^11 20^

Differences in patients’ characteristics may, at least in part, explain why RAPID3 seems to capture treatment effects differently in RCTs and observational cohorts. RAPID3 mostly relies on subjective assessments of disease activity, and these are potentially influenced by comorbidities that are less likely to occur in patients selected for RCTs. In addition, patients from trials usually have high levels of inflammation at study entry. In such setting, ‘subjective pain’ may be less relevant than ‘inflammatory pain’, and outcome measures to quantify disease activity will mostly reflect the latter. It should be noted, however, that our data stem from a single-centre cohort of patients on stable treatment and with limited change in disease activity over time. Limited change may preclude subtle associations to be detected (eg, between ‘subjective’ and ‘objective’ pain). Larger studies in patients with higher levels of, and most importantly, larger changes in disease activity (measured also by scores other than DAS28) should give resolution.

Taken all together, these data suggest that RAPID3 is most likely not suitable to be used alone for monitoring disease activity in patients with RA in daily clinical practice, since important objective information on the presence of inflammation (acute phase reactants and swollen joints) is missed. For instance, if subjective pain is driving high scores in a patient and this leads to treatment intensification (eg, changing or adding disease-modifying antimodifying antirheumatic drugs), the end result might be ‘overtreatment’, which carries negative consequences both to the patient and to the society.^21^ A so-called ‘dual strategy’ has been proposed by Ferreira *et al*,^22^ which defends the use of PROMS as complementary to evaluate the global impact of disease beyond inflammatory-driven disease activity in order to define additional needs for care. This approach still needs to be proven in practice.

In conclusion, our results show that DAS28 and RAPID3 do not associate with each other in patients with RA from daily clinical practice. RAPID3 is strongly driven by (subjective) ‘pain’, and its sole use as a monitoring instrument to guide treatment decisions can be misleading and potentially harmful to patients.
