# Supplementary material for: Routine Assessment of Patient Index Data 3 (RAPID3) alone is insufficient to monitor disease activity in rheumatoid arthritis in clinical practice
Source: RMD Open. 2019 Nov 28;5(2):e001050. doi: 10.1136/rmdopen-2019-001050 (PMC6927511; doi:10.1136/rmdopen-2019-001050)
Supplement: Supplementary data [file rmdopen-2019-001050supp001.pdf]

Routine Assessment Patient Index Data 3 (RAPID3) alone is insufficient to monitor disease activity in rheumatoid arthritis in clinical practice

SUPPLEMENTARY MATERIAL

Online Supplementary Text S1: Details on statistical analysis

Step 1: Observed change over time

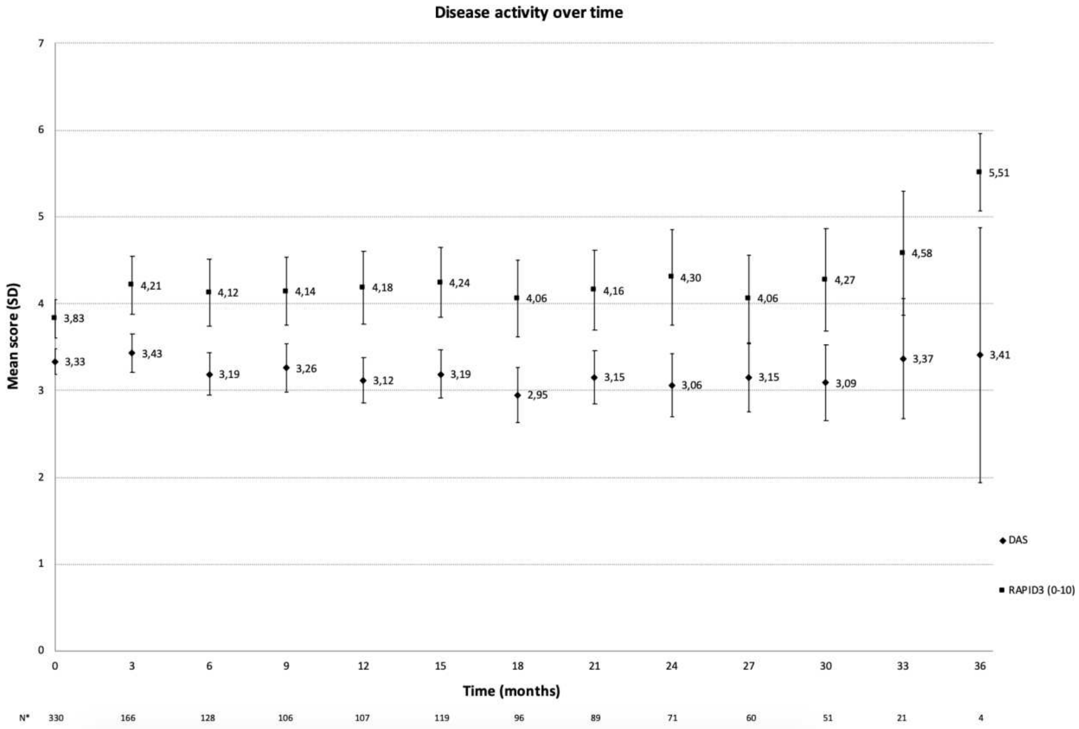

Supplementary Figure 1 Mean score (SD) of DAS28 and RAPID3 (0-10) over time. DAS28 ESR, 28 Joint Disease Activity Score (four variables, ESR-based); RAPID3 (0-10), Routine Assessment of Patient Index Data (3 domains; functioning, pain, patients` global).
*Number of patients per visit.

Step 2: Correlation structure

- Pairwise Spearman correlation coefficients were calculated for repeated DAS28 and RAPID3 (0-10) observations over time. Correlation coefficients ranged from 0.4 to 0.8 and 0.7 to 0.9 for DAS28 and RAPID3 respectively.

- The exchangeable working correlation structure (approximately equal correlation over time) was found to best fit the data and was used in all generalised estimating equations (GEE) -models.

Step 3: Linearity assumption testing

- **Linear model:**
  - DAS28: quasi-likelihood under the independence model criterion (QIC): 2872.673
  - RAPID3 (0-10): QIC: 56116.859
- **Distributions other than linear:**
  - DAS28:
    - Quadratic factor: QIC: 2873.706; p-value: 0.530
    - Cubic factor: QIC: 2873.208; p-value: 0.427
  - RAPID3 (0-10):
    - Quadratic factor: QIC: 56188.282; p-value: 0.480
    - Cubic factor: QIC: 56102.317; p-value: 0.677

- The lower the QIC the better the model fit. Linear (i.e. the simplest) distribution was assumed since neither the quadratic nor the cubic distribution proved to fit the data better (supplementary Figure 2).

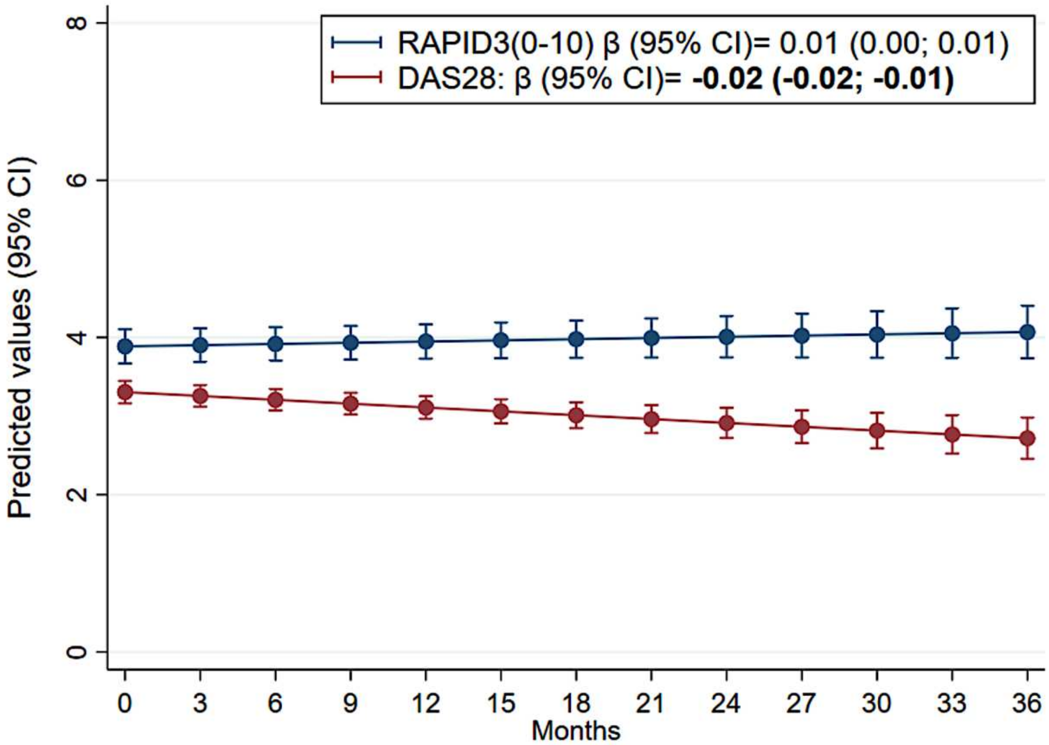

Supplementary Figure 2 Predicted values (95% CI) of DAS28 and RAPID3 (0-10) over time using linear GEE -models. DAS28 ESR, 28 Joint Disease Activity Score (four variables, ESR-based); RAPID3 (0-10), Routine Assessment of Patient Index Data (3 domains; functioning, pain, patients` global); GEE, Generalized estimating equation.

Step 4: GEE -model using auto-regression

- All models were fitted adjusting for the outcome t-1 to disentangle the within-patient from the between-patient effect.
- The model reflecting the longitudinal association between RAPID3(0-10) and DAS28 is represented by equation 1.
- The models describing the longitudinal association between RAPID3(0-10) and the individual DAS28 components are depicted by equations 2-5.

Equation 1: $\mu_i = \beta_0 + \beta_1 * RAPID3(0\text{-}10)_i + \beta_2 DAS28\_t\text{-}1_i + e_i$

Equation 2: $\mu_i = \beta_0 + \beta_1 * RAPID3(0\text{-}10)_i + \beta_2 SJC\_t\text{-}1_i + e_i$

Equation 3: $\mu_i = \beta_0 + \beta_1 * RAPID3(0\text{-}10)_i + \beta_2 TJC\_t\text{-}1_i + e_i$

Equation 4: $\mu_i = \beta_0 + \beta_1 * RAPID3(0\text{-}10)_i + \beta_2 ESR\_t\text{-}1_i + e_i$

Equation 5: $\mu_i = \beta_0 + \beta_1 * RAPID3(0\text{-}10)_i + \beta_2 PGA\_t\text{-}1_i + e_i$

μ_i= population-average DAS28 ESR (or individual DAS component)
i= index patient
β_0= intercept
β_1= association between RAPID3 (0-10) and DAS28 ESR (or individual DAS component)
β_2= auto-regressor: DAS-28 ESR (or individual DAS component) in t-1
e_i = residual error
